# Chronic Musculoskeletal Disabilities following Snake Envenoming in Sri Lanka: A Population-Based Study

**DOI:** 10.1371/journal.pntd.0005103

**Published:** 2016-11-04

**Authors:** Subashini Jayawardana, Ariaranee Gnanathasan, Carukshi Arambepola, Thashi Chang

**Affiliations:** 1 Department of Allied Health Sciences, University of Colombo, Colombo, Sri Lanka; 2 Department of Clinical Medicine, University of Colombo, Colombo, Sri Lanka; 3 Department of Community Medicine, University of Colombo, Colombo, Sri Lanka; Faculty of Medicine, University of Kelaniya, SRI LANKA

## Abstract

**Background:**

Snakebite is a major public health problem in agricultural communities in the tropics leading to acute local and systemic impairments with resultant disabilities. Snakebite related long-term musculoskeletal disabilities have been a neglected area of research. We conducted a population-based, cross-sectional study in an agricultural community to describe the chronic musculoskeletal disabilities of snake envenoming.

**Methodology/Principal Findings:**

A sample representative of residents of a single district in a region of high incidence of snake envenoming was recruited to identify ever snakebite victims. They were evaluated for chronic musculoskeletal disabilities that had developed immediately or within four weeks after the snakebite and persisted over three months. In-depth interviews, validated musculoskeletal functional assessment criteria and specialists’ examinations were utilised. Among the 816 victims, 26 (3.2%, 95% confidence interval: 2.2–4.6%) had musculoskeletal disabilities, persisting on average for 13.4 years (SD = 14.4). The disabilities were mostly in lower limbs (61.5%) and ranged from swelling (34.6%), muscle wasting (46.1%), reduced motion (61.5%), reduced muscle power (50%), impaired balance (26.9%), chronic non-healing ulcers (3.85%), abnormal gait (3.85%), fixed deformities (19.2%) to amputations (15.4%). Based on disability patterns, six snakebite-related musculoskeletal syndromes were recognised. The offending snakes causing disabilities were cobra (30.8%), Russell’s viper (26.9%) and hump-nosed viper (7.7%). Cobra bites manifested muscle wasting (87.5%), reduced muscle power (87.5%), joint stiffness (62.5%) and deformities (37.5%) while viper bites manifested impaired balance (42.8%), pain (71.4%) and swelling (71.4%).

**Conclusions/Significance:**

Snakebite envenoming is associated with considerable long-term musculoskeletal disabilities. Facilities for specialized care and rehabilitation need to be established in high risk areas.

## Author Summary

Snakebite is a neglected tropical disease mainly affecting rural agricultural communities in tropical and subtropical countries. The acute effects of snake envenoming have been widely studied and well characterized, but the long-term sequelae remain poorly characterized. Given the known acute effects of snake venom on the musculoskeletal system, we undertook a population-based study to assess the long-term musculoskeletal sequelae including disabilities in an agricultural community in Sri Lanka, which is a country with a high incidence of snakebites. Among 816 victims of snake envenoming in a single district, we found 26 (3.2%) to have musculoskeletal disabilities persisting for months to years since the snakebite. The disabilities were mostly in the lower limbs and ranged from swelling, muscle wasting, stiff joints, reduced muscle power, impaired balance, fixed deformities, chronic non-healing ulcers, long-standing lumps to limb or digit amputations. Cobra envenoming accounted for a third of these disabilities while another third was related to Russell’s and hump-nosed viper bites; the snake was not identified among the rest. Our study provides population-based data on the long-term musculoskeletal disabilities of snakebites and highlights the need for early institution of specialised care and rehabilitation in regions where snakebite is a public health problem.

## Introduction

Snake envenoming is a serious public health problem among people who live in tropical and sub-tropical regions of the world. As many as 1.2 million snakebites, 180,000 health sequelae and more than 60,000 deaths are reported annually in South Asia [1].

Sri Lanka, which is a tropical island positioned between Northern latitudes 5° and 10°, provides shelter to 105 terrestrial species of snakes. Among these snakes, six are medically important as venomous: Indian cobra (*Naja naja*), Common and Sri Lankan kraits (*Bungarus caeruleus* and *B*. *ceylonicus*), Russell’s viper (*Daboia russelii*), Saw-scaled viper (*Echis carinatus*) and Hump-nosed pit viper (*Hypnale hypnale*) [2]. Sri Lanka is one of the highest envenoming reporting countries in the region with an estimated incidence of over 80000 bites, 30000 envenomings and 400 deaths per year, predominantly among rural residents engaged in agriculture related occupations [3].

Snake venom contains more than 20 different constituents—mainly proteins including enzymes and polypeptide toxins. Cytolytic or necrotic toxins are digestive hydrolases (proteolytic enzymes and phospholipases A). Polypeptide toxins and other factors increase permeability resulting in local swelling. They may also destroy cell membranes and tissues. Haemolytic and myolytic phospholipase A2 enzymes damage cell membranes, endothelium, skeletal muscle, nerves and red blood cells [4]. These effects mediated by snake venom toxins in the acute phase have been widely studied and well characterised in practice as clinical syndromes [2]. However, the long term sequelae of these toxins that may result in disabilities have not been studied to the same extent [5].

The International Classification of Functioning, Disability and Health (ICF) defines disability as an umbrella term for impairment, activity limitation and participation restriction [6] Disability following snakebite is not an uncommon entity in tropical countries. Chronic kidney disease, visual loss, myocardial ischaemia, foetal loss, stroke, depression and post-traumatic stress disorder are some of the well-known long term disabilities of systemic envenoming [2,7,8,9] In contrast, disabilities owing to local envenoming are mostly encountered in the acute stage as immediate consequences, but whether venom toxins could cause protracted sequelae remains unknown. It is likely that local tissue necrosis, amputation, arthrodesis, etc. involving the locomotor apparatus may lead to disabilities in the musculoskeletal system as late consequences of local envenoming. On the other hand, such chronic disabilities may accrue as a result of inadequate medical intervention in the long term and lack of rehabilitation therapy following snakebite. Chronic disability, if found to persist long after snakebite, would highlight the need for secondary prevention in snake envenoming. Thus, this study aimed to characterise the long-term musculoskeletal related disabilities and how such disabilities are related to their health seeking behaviour and daily life in a rural population plagued with the occupational hazard of frequent snake bites.

## Methods

### Study setting and population

A population-based cross-sectional study was conducted in Ampara, the largest of the three districts in the Eastern province of Sri Lanka, which comprises a land area of 4,415 km^2^ with nearly 200 km^2^ of water sources and natural forests, and is divided into administrative divisions constituting 20 Divisional Secretariat (DS) divisions and 508 Grama Niladhari (GN) divisions. The main livelihood of its people is paddy, sugar-cane and chena cultivation. Such outdoor vocations make the residents highly vulnerable to snakebites while their delayed access to health care facilities due to the rough terrain and remoteness of the area worsen the outcomes.

The study population consisted of residents living in the district of Ampara for more than one year who had been recruited for a large population-based prevalence study on snakebite. Semi-permanent residents in commercial/work sites, permanent residents in religious institutes or elderly homes, and infants were excluded from the study. The sample size calculated for the prevalence study was 8470 residents, to detect an expected prevalence of 0.04 (based on a pilot survey conducted in the same district) with precision of 0.015; 5% level of significance; design effect of 10.8 calculated for an intra-class correlation of 0.2 and cluster size of 50; and 15% non-response. To obtain this sample, a multi-stage sampling method was used within the framework of administrative divisions of the district to select 2500 households from 10 randomly selected DS divisions. Within each DS division, five GN divisions were randomly selected. Within each GN division, 50 households were systematically selected. Within each household, all eligible members belonging to the nuclear family were recruited. If the participant was less than 12 years of age, the parent/guardian was interviewed. To minimise non-response, data collectors visited the households during week days while avoiding the farming hours and second visits (if needed) were made during weekends. If an eligible person was not available for recruitment after two visits to the household, he/she was considered a non-respondent in the study.

### Data collection

Data collectors comprised two graduates in Physiotherapy who were well-trained in data collection techniques. A pre-tested questionnaire in the local vernacular languages (Sinhala and Tamil) was administered to all study participants to obtain data on socio-demographic characteristics and evidence on snake envenoming. A ‘snakebite victim’ was defined as any person who had been bitten by a snake during his/her lifetime. Documental evidence kept with the participants such as discharge summaries, diagnosis cards and clinic records (which are usually given to the patient for safekeeping in Sri Lanka) was used to corroborate snakebites.

Assessment of chronic musculoskeletal disabilities following snakebite was done objectively based on history, examination and application of validated musculoskeletal functional assessment criteria. Initially, a detailed history was obtained from each snakebite victim in an interview with open-ended questions on symptoms related to the musculoskeletal system such as pain, swelling, change in appearance, movement limitations and altered sensations that they had developed in the bitten limb immediately or within four weeks after the bite, and which had persisted for more than three months at the time of data collection. Among those who had such symptoms, disabilities were then assessed and confirmed according to the operational definition of WHO of ‘having any restriction or lack of ability to perform an activity in the manner or within the range considered normal for a human being’ [4]. These assessments were done by the two Physiotherapists independently and included clinical observations for fixed deformities and abnormalities in posture and gait; palpation for tenderness and local swelling; and a detailed examination of the affected limb for range of motion of joints, muscle power, static and dynamic balance abilities, and gait analysis. Range of movement was assessed using a goniometer; muscle power using handheld dynamometers for grip strength and manual muscle testing scale for other muscle groups [10]; and standing balance using functional reach test [11]. All the instruments were calibrated prior to measurement and operated using a detailed interviewer guide/manual. Subsequently, in order to confirm the disabilities, all symptomatic snakebite victims were further examined by a Specialist physician and a Specialist neurologist who recorded their findings independently. Participants were re-examined if there were discrepancies between the findings of the examiners and consensus conclusions were made. Nerve conduction tests were applied whenever neurological examination findings were found to be present. Finally, information of the impact of these disabilities on daily life and health seeking behaviour including rehabilitation that they had undergone was obtained. Data of all study participants were obtained during house to house visits, and from among them, those participants in whom disabilities were detected were requested to visit a hospital/location close to their residence for further confirmation by the two Specialists.

### Statistical methods

Data were analysed using statistical package for social sciences (SPSS) version 20. Prevalence of chronic musculoskeletal disabilities was estimated in percentage and 95% confidence intervals (CI). Characteristics of the snakebite victims were described in mean and standard deviations (SD) for quantitative data and in proportions for qualitative data. Significant differences between the victims with and without musculoskeletal disabilities in relation to their socio-demographic characteristics were assessed using chi-square test (or Fisher’s Exact test, whenever the expected count of any cell was less than 5).

### Ethics statement

Ethical approval for the study was given by the Ethics Review Committee of the Faculty of Medicine, University of Colombo, Sri Lanka. All interviews were conducted after obtaining informed written consent. Permission for conducting the study was obtained from district and divisional level public administrators before data collection. Administrative officers of the sampled GN divisions were pre-informed about the study, which was conducted with their cooperation. No animals were used in the study.

## Results

Of the 8,707 residents recruited into the study, 816 have had a snakebite at least once during their lifetime. The snakebite victims belonged to a diverse ethnic distribution (Sinhalese 76.6%; Tamils 8.7%; Muslims 10.3%; other ethnicities 4.4%) and 60% of them were males. Their mean age was 42.7 years (SD = 16.3).

### Prevalence of chronic musculoskeletal disabilities following snakebite

Of the 816 snakebite victims identified, 26 (3.2%; 95% CI: 2.2–4.6%) were found to have musculoskeletal disabilities involving their bitten limb that had persisted for an average period of 13.4 years (SD = 14.4) since the bite, ranging from 3 months to 55 years with a median duration of 10 years. These disabilities involved the upper limbs in 10 (38.5%) and the lower limbs in 16 (61.5%).

### Characteristics of snakebite victims with chronic musculoskeletal disabilities

Table 1 shows the socio-demographic characteristics of victims with chronic musculoskeletal disabilities compared to those without disabilities. The majority of disabled victims were females (53.8%); of Sinhalese ethnicity (61.5%); married (84.6%); educated up to Grade 5 (50%); and earning below Rs. 20,000 (92.3%). Their mean age was 46.8 years (SD = 19.1) compared to 42.6 years (SD = 16.1) among those without disabilities, and were scattered in all 10 DS divisions. None of these characteristics were significantly different from those who did not develop any disability (p>0.05).

**Table 1:** Characteristics of all snakebite victims and those with chronic musculoskeletal (MSK) disabilities following snakebite.

Characteristic	Snakebite victims		Significance
	With MSK disabilities	Without MSK disabilities	
**Ethnicity**			
Sinhala^1^	16 (61.5%)	609 (77.1%)	X^2^ = 3.5
Tamil^2^	4 (15.4%)	67 (8.5%)	df = 1
Muslim^2^	4 (15.4%)	80 (10.1%)	p = 0.32
Other^2^	2 (7.7%)	34 (4.3%)	
**Gender**			X^2^ = 2.16
Male	12 (46.2%)	478 (60.5%)	df = 1
Female	14 (53.8%)	312 (39.5%)	p = 0.14
**Age group**			
1–19 years	0 (0.0%)	28 (3.5%)	X^2^ = 1.7
20–39 years	14 (53.8%)	474 (60.0%)	df = 2
40 years and above	12 (46.2%)	288 (36.5%)	p = 0.42
**Current marital status**			
Never married^1^	4 (15.4%)	113 (14.3%)	X^2^ = 0.22
Married^2^	22 (84.6%)	671 (84.9%)	df = 1
Separated/divorced/widow^2^	0 (0.0%)	6 (0.7%)	p = 0.97
**Highest level of education**			
No formal education^1^	1 (3.8%)	54 (6.8%)	X^2^ = 0.03
Grade 1–5^1^	12 (46.2%)	327 (41.4%)	df = 1
Grade 6–10^2^	12 (46.2%)	277 (35.1%)	p = 0.86
Passed Ordinary Level Examination ^2^	1 (3.8%)	117 (14.8%)	
Passed Advanced Level Examination^2^	0 (0.0%)	14 (1.7%)	
Higher education^2^	0 (0.0%)	1 (0.1%)	
**Current occupation**			
Employed^1^	17 (65.4%)	597 (75.6%)	X^2^ = 1.4
Student^2^	2 (7.7%)	65 (8.2%)	df = 1
Housewives^2^	5 (19.2%)	104 (13.2%)	p = 0.24
Unemployed^2^	2 (7.7%)	24 (3.0%)	
**Total**	**26 (100.0%)**	**790 (100.0%)**	

^1, 2^ Categories have been combined due to small numbers when applying statistics

**Fig 1:**
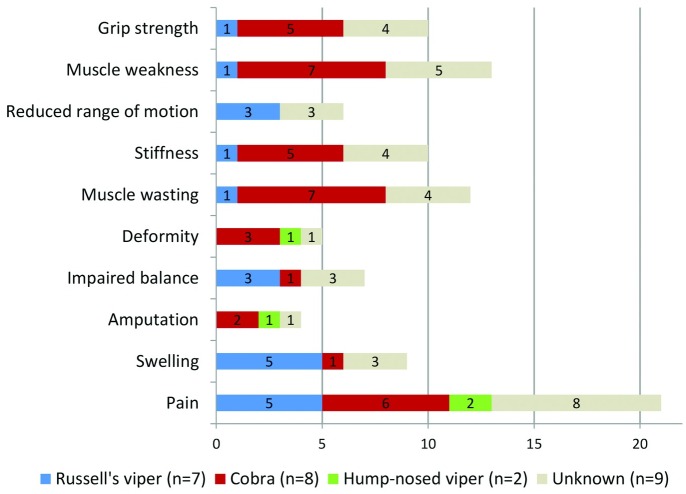
Distribution of the snakebite victims according to their presenting clinical symptoms.

**Fig 2:**
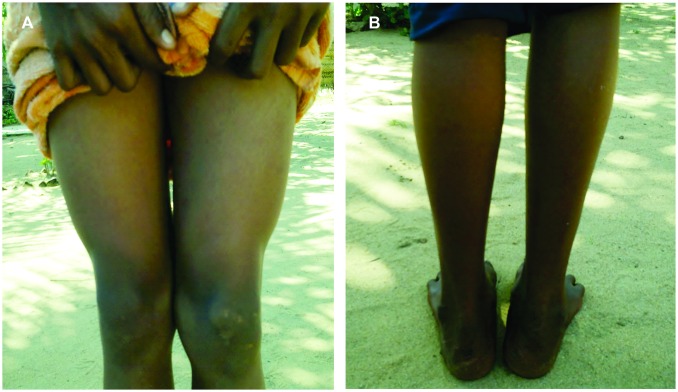
Wasting of right quadriceps muscles (A) and wasting of right calf muscles (B) following cobra bite.

**Fig 3:**
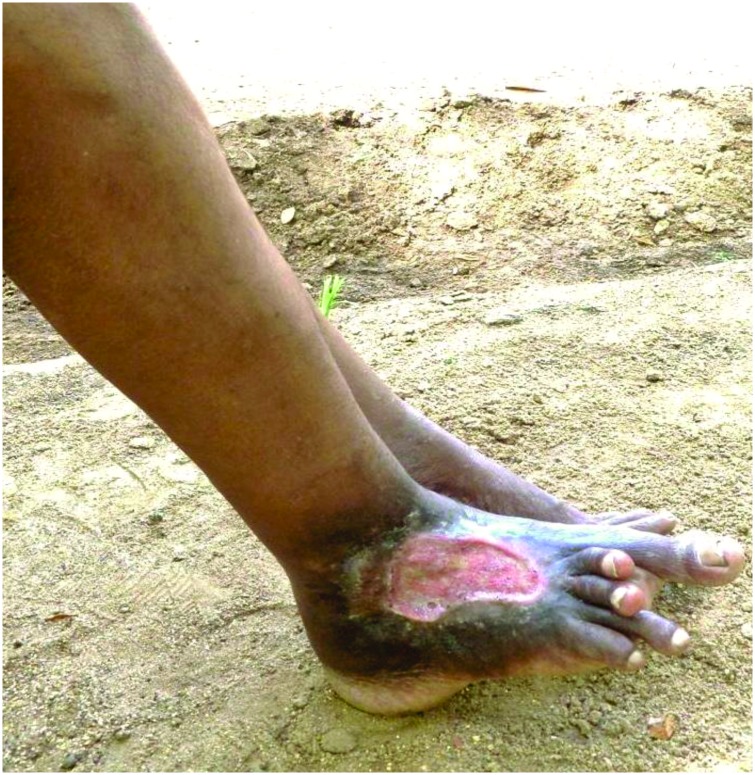
Chronic non-healing ulcer of the right foot and fixed deformities of toes following cobra bite.

**Fig 4:**
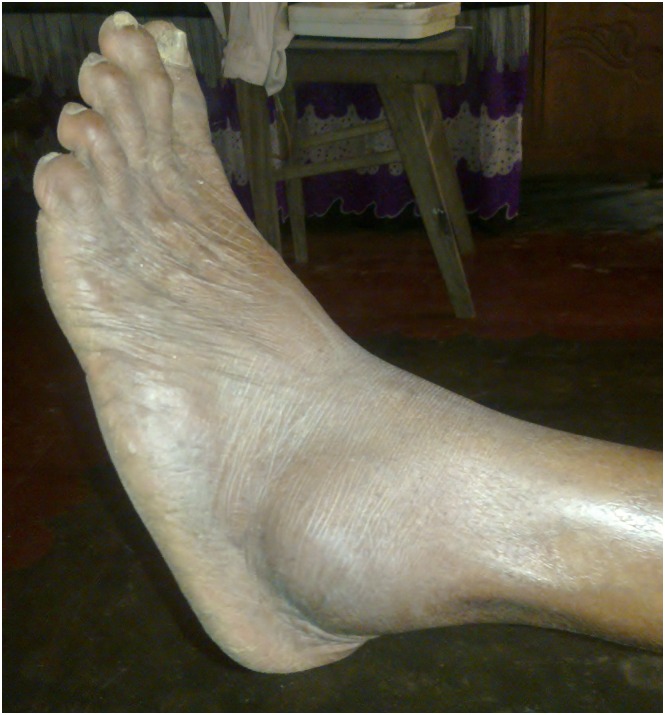
Persistent lump over the lateral malleolus (arrow) following viper bite.

**Fig 5:**
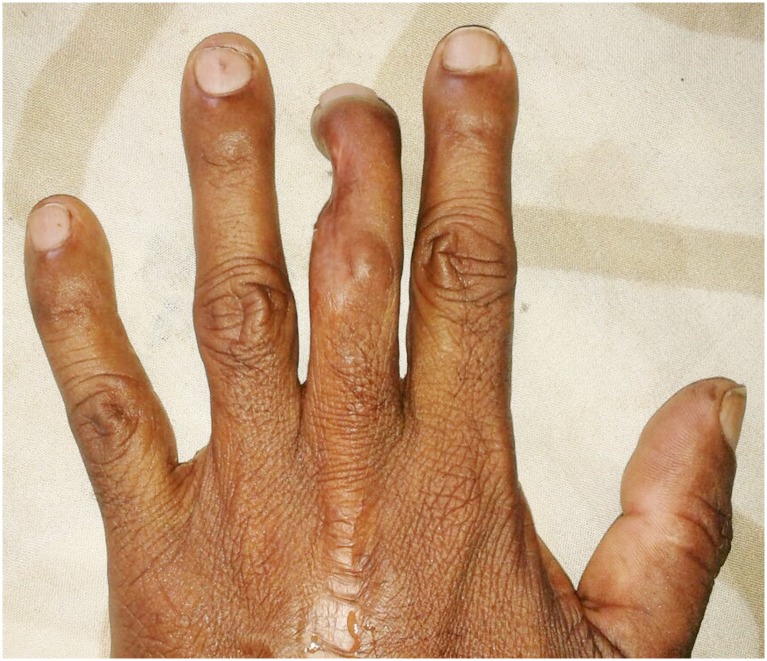
Fixed finger deformities in the left hand following snakebite.

**Fig 6:**
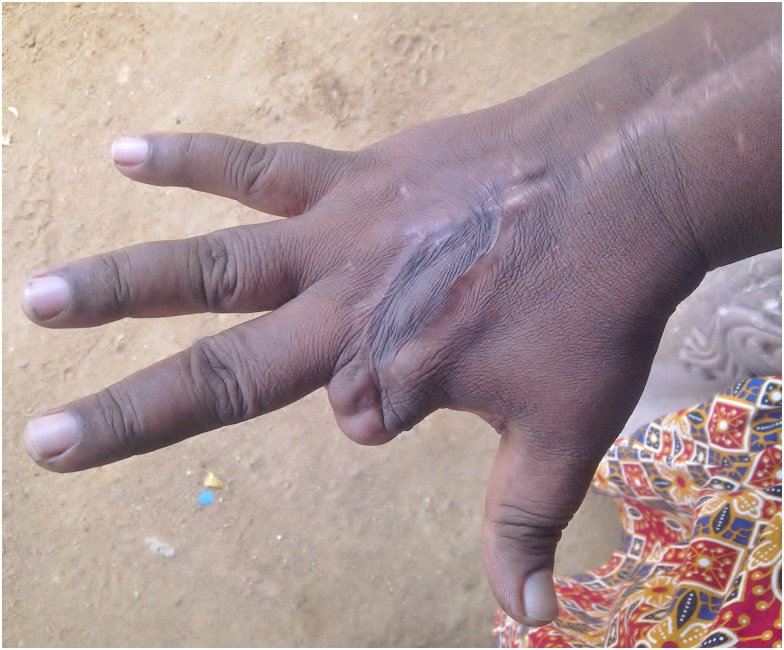
Amputation of the right index finger following cobra bite.

Detailed clinical and snakebite related characteristics of the victims who developed musculoskeletal disabilities are shown in Fig 1 and Table 2. At the time of data collection, the commonest presenting complaint was local pain in the affected limb (n = 21, 80.8%). On general examination, nine victims (34.6%) had swelling of the affected limb while 12 (46.1%) showed apparent muscle wasting over gastrocnemius, soleus and quadriceps (Fig 2A and 2B), and flexor compartment of the upper limb and small muscles of hands. One victim (3.85%) presented with a chronic non-healing ulcer (Fig 3) and another with a long-standing lump on the bitten site (Fig 4). On further examination, reduced muscle power was noted in 13 (50.0%), reduced range of motion in 16 (61.5%) and balance impairment in 7 (26.9%). Only one victim (3.85%) demonstrated an abnormal gait pattern. Severe forms of musculoskeletal complications included fixed deformities (5, 19.2%) (Figs 3 and 5) and amputation of the bitten limb/digits (4, 15.4%) (Fig 6).

**Table 2:** Clinical presentations of musculoskeletal disabilities following snakebite.

Musculoskeletal disability				Patient characteristics					
Presenting complaint	On examination	Site	Snake^a^	No.	Duration since bite	Circumstance	Sex	Age (yrs.)	Occupation
**(A) Intermittent pain over foot**	Chronic ulcer Ankylosed ankle joint Wasting and weakness of quadriceps, hamstrings, gastrocnemius and soleus muscles Soft tissue Hypertrophy at the heel Foot deformity	Foot	C	1	34 years	While walking	F	47	Farmer
**(B) Intermittent swelling over ankle**	Lump over ankle joint No tenderness	Ankle	RV	2	31 years	While walking	F	72	Housewife
**(C) Pain during prolonged standing and walking**	Wound Scar Limited ankle dorsiflexion Wasting and weakness of quadriceps, gastrocnemius and soleus muscles Impaired standing balance	Foot	C	3	2 years	While standing	F	16	None
			NI	4	5 years	While walking	F	61	House wife
	Partial amputation Impaired standing balance	Big toe	HNV	5	2 years	While walking	F	40	Manual labourer
	Tenderness over tendoachilles Limited ankle plantar flexion Wasting and weakness of gastrocnemius, quadriceps and soleus muscles Impaired standing balance	Achilles tendon	RV	6	4 months	During harvesting	M	65	Farmer
				7	15 years	While farming	M	42	Farmer
			C	8	2 years	While playing	M	16	Student
			NI	9	12 years	While sleeping	M	19	Unemployed
				10	13years	While standing	F	28	Housewife
**(D) Intermittent swelling and pain of ankles**	Ankle swelling Limited ankle dorsiflexion Impaired standing balance	Above ankle	RV	11	7 months	While walking	M	33	Farmer
		Ankle		12	8 years	While walking	F	27	Housewife
				13	3 months	While walking	M	66	Farmer
		Ankle	NI	14	9 years	While walking	F	34	Housewife
				15	12 years	While walking	F	27	Housewife
				16	10 years	While walking	M	63	Farmer
**(E) Manual disability**	Amputated right index finger Reduced grip strength Weakness and wasting of upper limb muscles	Index finger	C	17	13 years	While reaching for an object from a wrack	F	45	Housewife
	Amputated right ring finger Reduced grip strength Weakness and wasting of upper limb muscles	Thumb		18	10 years	While sleeping	F	73	Farmer
	Amputated right ring finger Reduced grip strength Weakness and wasting of upper limb muscles	Ring finger	NI	19	55 years	While putting the hand in to a anthill	F	60	Housewife
**(F) Pain in upper limb caused by prolonged or repetitive use**	Stiff interphalangeal joints Reduced grip strength	Thumb	C	20	20 years	While working in the chena	M	63	Farmer
		Index finger	RV	21	17 years.	While working in the field	M	37	Farmer
		Ring finger	NI	22	50 years.	While collecting firewood	F	65	Housewife
	Reduced grip strength Stiff interphalangeal joints Wound scar Wasting of hypothenar muscles and forearm flexor muscles Muscle contractures Finger deformity	Ring finger	NI	23	4 months	While cutting firewood	M	27	Farmer
		Ring finger	HNV	24	10 years	While collecting firewood	M	73	Farmer
		Middle finger	C	25	6 years	While working in the garden	M	50	Farmer
		Index finger		26	10 years	While collecting firewood	F	60	Housewife

^a^ C = Cobra; RV = Russell’s viper; HNV = Hump nosed viper; NI = Not identified

Among the 26 victims who had disabilities following snakebite, the offending snake could be confirmed only in 17 (65.4%). Among them, the offending snake was identified as cobra (8, 30.8%), Russell’s viper (7, 26.9%) and hump-nosed viper (2, 7.7%). The majority of cobra bites showed muscle wasting (7, 87.5%), reduced muscle power (7, 87.5%), joint stiffness (5, 62.5%), and deformities (3, 37.5%). In contrast, Russell’s viper bites demonstrated impaired balance (3, 42.8%), pain (5, 71.4%) and swelling (5, 71.4%) in the affected limb.

Six musculoskeletal syndromes were recognised among the snakebite victims (Table 2): (A) intermittent pain in the bitten limb associated with ankylosed joint adjacent to the bite and wasting and weakness of regional muscle groups; (B) intermittent swelling of the bitten limb associated with lumps but no pain or tenderness; (C) pain during prolonged standing and walking associated with limitation of range of movement of ankle joint, wasting and weakness of regional muscle groups and impaired standing balance; (D) intermittent pain and swelling of the bitten limb associated with joint swelling, limitation of range of movement of ankle joint and impaired standing balance; (E) manual disability following upper limb bites associated with amputation of digits, reduced grip strength and wasting and weakness of regional muscle groups; (F) pain with repetitive or prolonged use following upper limb bites associated with flexion deformities, reduced grip strength and wasting and weakness of regional muscle groups.

It is notable that some of these musculoskeletal syndromes had persisted for over a decade after the snakebite. A chronic non-healing ulcer associated with foot deformity was noted in a patient (Fig 3), 34 years after a cobra bite despite repeated attempts at skin grafting. A biopsy of the ulcer done during this study excluded malignant transformation while skeletal and soft tissue imaging excluded chronic osteomyelitis.

Following the snakebite, all victims had sought Western treatment within the first 3 hours of the bite. Soon after the bite, the majority have shouted for help and were brought on motor bicycles or three-wheeled taxis (‘tuk-tuks’) to the hospital, while a few had walked home or to hospital. After discharge from hospital, almost all had visited the local indigenous doctor for treatment of persisting symptoms, but none had attended any rehabilitation programmes for their musculoskeletal disabilities. The majority of snakebite victims were unaware as to whom to visit for their current disabilities.

Musculoskeletal disabilities have affected the daily life of all victims. Those with foot deformities were unable to wear footwear, run or squat. Those with muscle wasting found it difficult to work in the field, cook, lift heavy objects or perform activities requiring fine motor movements such as picking up a coin or writing. However, the majority of victims continued to work in their paddy fields despite these disabilities.

## Discussion

Snake envenoming has been reported to lead to a variety of musculoskeletal disorders such as amputation of limbs or digits, Volkmann contracture, joint stiffness, arthritis, disseminated osteomyelitis, chronic ulceration with malignant transformation, Raynaud phenomenon and loss of muscle mass [12–16]. However, population-based epidemiological studies of musculoskeletal disorders, possible causes and residual sequelae of snake envenoming are sparse [5,17,18]. Our study, which systematically screened over eight and a half thousand residents in a highly-vulnerable rural community for snakebites and its musculoskeletal complications, addresses this neglected area of research.

We found a prevalence of 3.2% for chronic musculoskeletal disabilities of snake envenoming among 816 snakebite victims among the 8,707 residents screened in an agricultural population plagued with the occupational hazard of snakebites. Two thirds were below the age of 60 years and most of them were involved in farming activities. Although the majority of snakebite victims were males, more females had developed musculoskeletal disabilities than males. The reason for a female predisposition for musculoskeletal disabilities is unclear.

Musculoskeletal complications in the acute phase following snakebite are not uncommon. These are often considered to be temporary effects of local trauma. Our study highlights the chronic musculoskeletal complications that had persisted for many years following the bite. Some of the victims had developed contractures and deformities, wasting of muscles, joint stiffness, reduced range of movement, impaired balance and disability due to limb/digit loss. Surprisingly, persistent local pain in the affected limb was the commonest presentation. This was thought to be due to peripherally generated neuropathic pain provoked by damage to peripheral nerve endings caused by venom toxin and trauma of the bite.

Most of the musculoskeletal disorders in our study were attributed to cobra, hump-nosed viper and Russell’s viper bites while krait bites appeared not to contribute to the chronic musculoskeletal complications. It is hypothesised that myotoxic and cytolytic constituents of cobra venom which causes severe tissue necrosis, and vasculotoxins in viper venom which causes endothelial damage are more likely to cause musculoskeletal damage than bungarotoxins of krait venom which interfere with neuromuscular transmission [19].

Severe local effects of envenoming causing muscle necrosis requiring limb or digit amputations have been reported mostly in relation to cobra bites [2]. In sub-Saharan Africa, the annual number of amputations due to snakebites reported ranged from 5,908 to 14,614 [14]. In our population-based study, only four amputations due to snakebites were noted among 816 victims (0.49%) over an average period of 13.4 years. Two were following cobra bites while one was following a hump-nosed viper bite. However, these victims have been rendered disabled due to the loss of grip ability and grip strength following amputations or contractures of fingers, particularly the thumb.

Of particular note is the complication caused by bites over the Achilles tendon. It is likely that such bites may have caused Achilles tendinitis leading to the reduced range of ankle movement and wasting of soleus and gastrocnemii muscles. Eccentric exercises have been reported to counteract the failed healing response that underlies tendinopathy by promoting collagen fibre cross-linkage within the tendon, thereby facilitating tendon remodeling [20–22]. However, none of our victims with Achilles tendinopathy had been instructed with such an exercise programme.

It was curious to note a chronic ulcer of 34 years following a cobra bite. Repeated attempts of skin grafting had failed suggesting permanent damage to the microvasculature and regenerative capacity of tissues following cobra envenoming. There was no histological evidence of malignant transformation in the ulcer or imaging evidence of chronic osteomyelitis.

It could be argued that some of the chronic disabilities noted may have been due to degenerative changes associated with aging. However, only a third of the snakebite victims with musculoskeletal disabilities were over the age of 60 years and the nature of disability such as focal wasting/weakness, digit amputation leading to loss of grip strength, isolated Achilles tendinitis, and chronic lumps/ulcers predominantly localised in the bitten limb makes this possibility less likely. Furthermore, the victims with musculoskeletal disabilities were not significantly different from the ones without, in their socio-demographic characteristics, including age.

Chronic musculoskeletal sequelae are likely to be related to the damaging effect of venom on tissues at and adjacent to the bite site than the effect of systemic envenoming. However, the dearth of studies on long term musculoskeletal sequelae, particularly from regions such as Latin America [23] and Africa [12, 14] plagued with snake species that cause severe local tissue damage, precludes confirmation of this postulation. Nonetheless, a need for therapies to counteract local venom effects has been recognised and used against some species [24].

None of the snakebite victims in our study had been provided with any long-term rehabilitation. Although most victims had sought Western medical treatment immediately after snakebite, many of them sought indigenous medical treatment following discharge from hospital. None were aware of the need for long term rehabilitation to prevent complications. This may be attributed to the fact that snakebite management largely emphasises treatment of systemic envenoming while local effects are neglected with no organised rehabilitation programmes for snakebite victims or patient education on long term complications.

Various factors may have led to permanent disabilities following snakebites. Three main factors were identified. Firstly, most of the victims were not aware about the need for rehabilitative therapies following snakebite. Secondly, due to their rural terrain and economic constraints, it was not possible to attend to the necessary follow-ups, which may have been further compounded by a three-decade-long civil conflict in the region. Thirdly, the lack of appropriate, potent and effective, indigenous antivenom may have resulted in inadequate treatment of the local tissue pathology which predisposed to chronic complications [25].

There are some limitations of this study. Since we considered an ever-bite, it depended on the individual’s memory, some spanning back several decades. However, since snakebite is a morbidly memorable event, bias introduced owing to long recall period is highly unlikely. Although the time lapse could have affected the identification of the snake, since patients in Sri Lanka are given discharge summaries/diagnosis cards and clinic medical notes for safe keeping, these were used to corroborate patient claims on the identity of the snake. Furthermore, living in these areas since childhood, rural residents are usually knowledgeable on identifying venomous snakes. The circumstances of the bite as well as the clinical manifestations, which were recorded in medical notes available with the patient, were also considered when confirming the identity of the snake. Our study is likely to be criticised for being limited to a single district of the 25 districts in Sri Lanka. However, this district had been selected because it represented a typical rural community predominantly engaged in outdoor agricultural occupations in Sri Lanka, located in a province with a high burden of reported snake bites. Thus, we feel that our data is representative of rural agricultural communities in the country. Ours was a cross-sectional study and did not include a comparison control group. However, since we operationalised the definition of chronic musculoskeletal disabilities as those disabilities that developed immediately or within four weeks after the snakebite and persisted for three months or longer to ensure that the disabilities were directly attributable to the snakebite, a comparison group was not deemed essential to interpret our data. While acknowledging that having a suitable analytical study design with an age- and sex-matched control group with no snakebite would have further enhanced the external validity of our findings, we feel that our data had robust internal validity and would provide the foundation for the formulation of hypothesis for such future case-control studies.

In conclusion, this study provides population-based data in a neglected area of research to suggest that snakebite envenoming is associated with considerable chronic musculoskeletal disabilities. It highlights the need to extend medical care beyond the acute phase of snakebite envenoming. Given that snakebites afflict mainly outdoor agricultural workers whose livelihoods are likely to be impeded by chronic musculoskeletal disabilities, this study advocates establishment of programmes to educate, screen, treat and rehabilitate chronic musculoskeletal disabilities related to envenoming in regions where snakebite is a public health problem.

## Supporting Information

S1 Checklist STROBE Checklist. (DOC)
